# Assessing Computational Steps for CLIP-Seq Data Analysis

**DOI:** 10.1155/2015/196082

**Published:** 2015-10-11

**Authors:** Qi Liu, Xue Zhong, Blair B. Madison, Anil K. Rustgi, Yu Shyr

**Affiliations:** ^1^Center for Quantitative Sciences, Vanderbilt University School of Medicine, Nashville, TN 37232, USA; ^2^Department of Biomedical Informatics, Vanderbilt University School of Medicine, Nashville, TN 37232, USA; ^3^Department of Medicine, Washington University School of Medicine, St. Louis, MO 63110, USA; ^4^Division of Gastroenterology, University of Pennsylvania Perelman School of Medicine, Philadelphia, PA 19104, USA; ^5^Department of Medicine, University of Pennsylvania Perelman School of Medicine, Philadelphia, PA 19104, USA; ^6^Abramson Cancer Center, University of Pennsylvania Perelman School of Medicine, Philadelphia, PA 19104, USA; ^7^Department of Cancer Biology, Vanderbilt University School of Medicine, Nashville, TN 37232, USA; ^8^Department of Biostatistics, Vanderbilt University School of Medicine, Nashville, TN 37232, USA

## Abstract

RNA-binding protein (RBP) is a key player in regulating gene expression at the posttranscriptional level. CLIP-Seq, with the ability to provide a genome-wide map of protein-RNA interactions, has been increasingly used to decipher RBP-mediated posttranscriptional regulation. Generating highly reliable binding sites from CLIP-Seq requires not only stringent library preparation but also considerable computational efforts. Here we presented a first systematic evaluation of major computational steps for identifying RBP binding sites from CLIP-Seq data, including preprocessing, the choice of control samples, peak normalization, and motif discovery. We found that avoiding PCR amplification artifacts, normalizing to input RNA or mRNAseq, and defining the background model from control samples can reduce the bias introduced by RNA abundance and improve the quality of detected binding sites. Our findings can serve as a general guideline for CLIP experiments design and the comprehensive analysis of CLIP-Seq data.

## 1. Background

RNA-binding proteins (RBPs) are the primary regulator of posttranscriptional gene expression [1]. As soon as RNAs are transcribed, they are associated with RBPs to form ribonucleoprotein (RNP) complexes. The RBP-RNA associations modulate the biogenesis, stability, cellular localization, and transport of the RNA and determine the fate and function of RNA molecules. Therefore, a high resolution and precise map of protein-RNA interactions is essential for deciphering posttranscriptional regulation under various biological processes.

CLIP (cross-linking and immunoprecipitation) is the main technology for studying protein-RNA interactions* in vivo* [2–4]. CLIP uses ultraviolet irradiation to form covalent crosslinks only at direct sites between RBP and RNAs* in situ*, followed by immunoprecipitation of the protein-RNA complex with an antibody specific to the RBP of interest. The copurified RNA fragments are amplified and sequenced and mapped back to the reference genome to reveal RBP binding sites. CLIP has been successfully applied to study protein-RNA interactions in bacteria, fungi, yeast, and mammals [4–10]. To obtain a more comprehensive view of protein-RNA interactions, recently, CLIP coupled with high throughput sequencing technology (CLIP-Seq or HITS-CLIP) [11–15] and several alternative approaches, such as photoactivatable ribonucleoside-enhanced CLIP (PAR-CLIP) [16–20] and individual nucleotide resolution CLIP (iCLIP) [21–26], has been developed. Compared with the low-throughput CLIP data, these approaches allow genome wide mapping of protein-RNA interactions and have demonstrated their power for a number of proteins [12–14, 27–34]. For example, in contrast to only 34 Nova-bound transcripts detected in the original CLIP experiments, the first application of HITS-CLIP identified 2,481 Nova-bound RNAs [12]. The genome-wide insights provide a robust and unbiased means to study RBP function and predict RBP action* de novo*, leading to a tremendous progress in these areas.

Given the large amount of data generated by CLIP-Seq, considerable computational effort is required to generate reliable quantitative information of protein-RNA interactions [35]. A series of computational steps is involved in CLIP-Seq analysis, including data preprocessing, reads mapping, peak calling, normalization and annotation, and motif discovery [35]. Although more approaches and tools have been developed to address the challenges of peak calling by considering the global and local background [36–40], there has been no clear consensus on the appropriate way to implement each computational step or the impact of a chosen step on the downstream analysis. For example, some studies used all reads to call peaks, while others employed reads after duplication removal thinking that reads mapped to the same location are due to amplification bias [12, 29, 34]. As another example, early studies simply used the read counts to rank peaks [12], while recent methods ranked the sites by the relative enrichment of CLIP-data to the average CLIP count within the transcript for RBPs binding pre-mRNAs or to individual gene expression for RBPs binding mRNAs, trying to correct the bias introduced by RNA abundance [28–30, 32, 41]. Here we performed a comprehensive evaluation of different strategies to preprocess the data, normalize the peaks, and choose background sequences in the motif discovery. We generated CLIP data for Lin28b in three different colon cancer cell lines (Caco-2, DLD1, and Lovo) and mouse colon tissues with input RNA or corresponding RNAseq as controls [31]. We compared different strategies on the accuracy of identifying LIN28B binding sites. Our findings can serve as a general guideline on the appropriate way to implement each computational step, which will enable the design of improved computational and experimental protocols for CLIP-Seq analysis to further investigate posttranscriptional regulatory networks.

## 2. Materials and Methods

### 2.1. CLIP, Input RNA, and mRNAseq

CLIP samples were prepared from Caco-2 cells (three replicates), DLD1, and Lovo cell lines (one replicate each) with a doxycycline-inducible LIN28B and colonic epithelia of Vil-Lin28b^Med^ mice (two replicates) using a modified CLIP-Seq protocol [31]. For crosslinking at 254 nm, cells were irradiated on ice using stratalinker 2400 (Stratagene). Immunoprecipitation was performed overnight at 4°C using anti-FLAG M2 magnetic beads (M8823, Sigma) for Flag-HA-tagged mouse or human LIN28B. RNase T1 digestion, adapter ligations, and RNP isolation were described in [31]. Caco-2 cells and colon epithelium were sequenced by Illumina Hiseq 2000 as multiplexed samples, while DLD1 and Lovo were each sequenced on a single lane.

**Figure 1:**
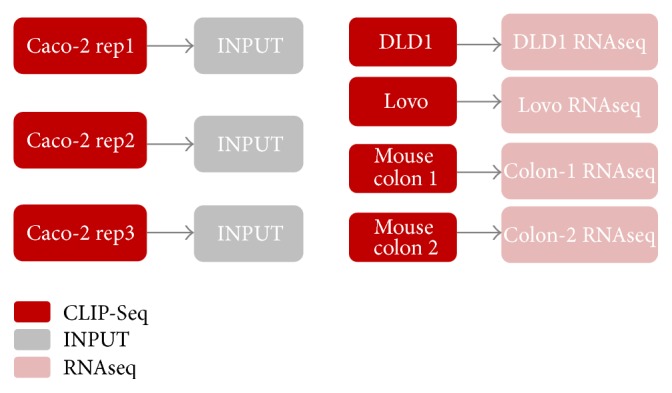
Experimental design of LIN28B CLIP-Seq. There are three replicates in CLIP Caco-2 samples with input RNA (no antibody) as control samples, one replicate each for DLD1 and Lovo CLIP samples with mRNAseq as control, and two replicates for mouse colon epithelium CLIP with mRNAseq as control.

We used two basic methods to produce control samples, input RNA, and mRNAseq. Caco-2 used input RNA as control samples, while DLD1, Lovo, and mouse colonic epithelium had mRNAseq (Figure 1). Input RNA samples were prepared from total RNA extracted from UV cross-linked Caco-2 cells by digestion for 30 minutes in Proteinase-K (Roche). RNA was depleted of ribosomal RNA using the RiboMinus Transcriptome Isolation Kit (K1550-02, Life Technologies) and then digested with 2 units of RNase-T1 (Fermentas). Total RNA samples from DLD1, Lovo, and mice colonic epithelium were depleted of ribosomal RNA and libraries were prepared using the NEBNext mRNA Library Prep Master Mix Set for Illumina (E6110S, New England Biolabs).

### 2.2. Reads Trimming and Mapping

CLIP reads for Caco-2 (50 bp), DLD1 (36 bp), Lovo (36 bp), and input RNA reads for Caco-2 (50 bp) were trimmed to remove adaptor sequences and mapped to human reference genome (hg19) using Novoalign (parameters: -*l* 18 -*t* 85 -*h* 90) (http://www.novocraft.com/), which require unambiguous mapping to the genome with ≤2 substitutions, insertions or deletions in ≥18 nt and homopolymer score ≥90. CLIP reads for mouse colonic epithelium (50 bp) were mapped to mouse reference genome (mm9) using Novoalign. mRNAseq reads for DLD1 and Lovo cell lines (101 and 100 bp) were mapped to human reference genome (hg19) using TopHat [42] and mRNAseq reads for mouse colonic epithelium (50 bp) were mapped to mouse reference genome (mm9) using Novoalign.

There were ~33–48 million reads for each CLIP Caco-2 sample and ~30% of reads could be uniquely mapped to the genome. In contrast, only ~12% of reads in input Caco-2 samples could be uniquely mapped to the genome, which was due to more severe adapter contamination. The percentage of pure adapter reads was much higher in input samples (~58%) than in CLIP samples (~25%) (Additional File 1 (see Supplementary Material available online at http://dx.doi.org/10.1155/2015/196082)). There were ~17–22 million reads for CLIP DLD1, Lovo, and mouse samples, ~200 million reads for DLD1 and Lovo RNAseq samples, and ~60 million reads for mouse colon RNAseq samples. About 20% of reads could be uniquely mapped to the genome for CLIP samples, while ~60% of reads could be uniquely aligned to the genome for RNAseq samples. The mapping results were summarized in Table 1. We also used BWA to map CLIP reads to the genome with default parameters and obtained lower percentage of aligned reads than Novoalign (data not shown here).

### 2.3. CLIP Peaks Calling and Normalization

CLIP peaks were called by HOMER (http://homer.salk.edu/homer/index.html) [43]. The global threshold for the number of reads that determine a valid peak was selected at a false discovery rate of 0.001 based on a Poisson distribution [43]. Peak sizes were chosen based on the length distribution of mappable reads.

It is known that CLIP-Seq read counts depend on the expression abundance of the corresponding transcript. To reduce the distortion introduced by sequencing bias or abundant RNA, normalization is recommended to make binding sites across the full transcriptome comparable [41]. Here we compared five different strategies to normalize and rank peaks: (1) no normalization, which simply ranks the peaks by the reads coverage (Raw); (2) normalizing to the average CLIP data, which ranks the peaks by the relative enrichment of CLIP counts to the average CLIP counts within the transcript (AVE-CLIP). This strategy is generally recommended to study RBPs binding pre-mRNA because it is difficult to measure the RNA abundance by the traditional RNAseq techniques; (3) normalizing to the average input RNA data, which ranks the peaks by the relative enrichment of CLIP counts to the average input counts within the transcript (AVE-INPUT); (4) normalizing to the input RNA, which ranks the peaks by the relative enrichment of CLIP counts to input counts within the same sites (INPUT); (5) normalizing to RNAseq (RPKM), which ranks the peaks by the relative enrichment of CLIP counts to the transcript abundance, obtained from RNAseq. Here RPKM (reads per kilobase of exon model per million mapped reads) was calculated to estimate the transcript abundance, where read counts were normalized by the transcript length as well as the total number of mappable reads. Using RNAseq as control sample is recommended and has proved to be useful in the analysis of RBPs targeting messenger RNAs (mRNAs) [29, 41].

### 2.4. Quality of Binding Sites

LIN28 is a conserved RNA-binding protein. It plays an important role in differentiation, reprogramming, and oncogenesis [44–47]. Mammals have two paralogs, Lin28a and Lin28b. In the previous studies, the motif GGAG has been reported as the binding site of Lin28a [48], which was also confirmed by crystal structure of mouse Lin28a in complex with pre-let7 families [49]. Although two recent CLIP-Seq experiments on Lin28a revealed different binding motifs, they both contained “GGAG” [27, 33]. Wilbert et al. found that Lin28 bound to GGAGA sequences [33], while Cho et al. reported that AAGGAG was the most frequently observed hexamers [27]. In addition, both of these two studies found that Lin28a-binding sites were enriched in exons but depleted of intronic regions, indicating that Lin28a largely interacts with messenger RNA. Lin28a and Lin28b have different physiological expression patterns but similar behavior* in vitro* [49]. Lin28b CLIP peaks were found mainly within mRNAs, with 70%~90% located in exonic regions [31]. The motif GGAG was detected in the binding sites of let-7 (Additional File 2).* De novo* motif analysis of robust CIMSs (cross-link induced mutation sites) from Caco-2 cells yielded the motif similar to GGAG [31]. Collectively, Lin28b, similar to Lin28a, binds messenger RNAs at the GGAG motif.

We used two criteria to assess the quality of peaks, the percentage of peaks located in exonic regions and the percentage of peaks containing the GGAG motif. The higher the percentage of exonic peaks and GGAG motif occurrence, the better the peak quality. Human exonic regions were obtained from Ensembl version 65. Mouse exonic regions were obtained from Ensembl version 61. Peaks that overlap with the annotated exonic reads at least 1 bp were counted as exonic peaks using BEDTools.

## 3. Results

### 3.1. Removing PCR Amplification Bias

**Figure 2:**
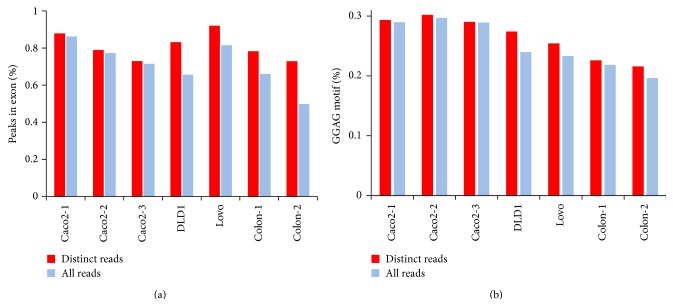
The percentage of CLIP peaks located in exonic regions and the GGAG motif occurrence when all reads or distinct reads were used.

PCR amplification artifacts distort the quantitative analysis of sequencing data. This problem is exacerbated in CLIP-Seq experiments whose library complexity is limited owing to numerous enzymatic steps required in the protocol and the small amount of starting material. One way to reduce amplification bias is duplication removal. To assess the effect of duplication removal, we compared the quality of peaks identified using either all unique mappable reads or distinct reads (reads mapped to same locations were collapsed) with the same threshold (FDR < 0.001). Compared to the method using all reads, using distinct reads yielded fewer peaks but a higher percentage of peaks in exonic regions for all CLIP samples (Figure 2). A slight increase in the percentage of exonic peaks was observed for Caco-2 CLIP samples using distinct reads versus all reads, while a larger increase was shown for DLD1, Lovo, and mouse colon samples, especially for DLD1 and Colon-2 CLIP samples (Additional File 3). For DLD1 CLIP samples, 83.2% of 17225 peaks were located in exonic regions using distinct reads. In contrast, only 65.6% of 65548 peaks were situated in exonic regions using all reads. Even if 16671 peaks were identified with a more stringent threshold, close to the number of peaks with distinct reads, only 79.8% (13311) were located in exonic regions. For mouse colon-2 CLIP samples, 72.9% of 6781 peaks were located in exonic regions using distinct reads, while only 49.8% of 13,302 peaks were found in exonic regions using all mappable reads. Furthermore, using distinct reads obtained higher percentage of peaks with GGAG motif compared to using all reads, especially for DLD1 and Lovo CLIP samples (Figure 2 and Additional File 3). The results suggest that using distinct reads reduces PCR amplification bias, leading to the improved quality of peaks.

### 3.2. Peak Normalization and Ranking

**Figure 3:**
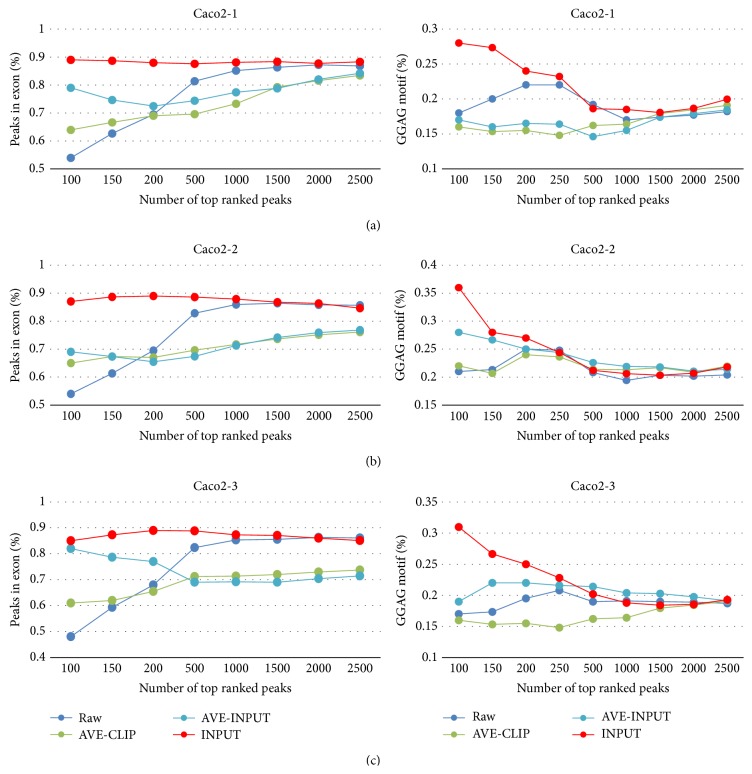
The percentage of peaks in exonic regions and the GGAG motif occurrence for Caco2 CLIP samples when four different normalization and ranking strategies were used, including Raw, AVE-CLIP, AVE-INPUT, and INPUT.

After peaks are identified, normalization is recommended to quantify protein-RNA interactions, making peaks across the transcriptome comparable. Here we assessed the performance of different peak normalization and ranking strategies. Important differences were observed between different methods. For Caco-2 CLIP samples with input RNA as control, we compared four different ranking and normalization strategies, including Raw, AVE-CLIP, AVE-INPUT, and INPUT (Materials and Methods). We estimated their performance by the percentage of peaks located in exonic regions and the GGAG motif occurrence among the top ranked peaks. The higher the rank is, the more reliable the peaks should be. That is, the accuracy is supposed to decrease as the number of top ranked peaks increases. However, the method without normalization (Raw) got lower percentage of exonic peaks and GGAG motif occurrence among more highly ranked peaks (Figure 3). For example, there were only 54% of peaks located in exonic regions among the top 100 peaks for the CLIP Caco-2 replicate 1 sample, in contrast to 70% in the top 200 peaks and 87.5% in the top 3000 peaks. Similarly, only 18% of peaks contained the GGAG motif in the top 100 peaks, compared to 22% in the top 200 peaks (Figure 3). These results suggest that there are lots of nonspecific and background binding in the highly ranked peaks. AVE-CLIP, although recommended by previous studies, did not show good performance and it even had the lowest percentage of the GGAG motif occurrence compared to the other three methods (Figure 3), indicating that averaging the CLIP-data on the individual transcript is not an appropriate way to reduce the bias introduced by the transcript abundance but instead weakens the signal. Input sample as control helped remove false positives. Normalizing to AVE-INPUT obtained higher percentage of exonic peaks and GGAG motif occurrence in the highly ranked peaks than the “Raw” method (Figure 3). However, it performed worse when the number of top ranked peaks increased, suggesting that it distorts the binding affinity when the signal turns weaker. Normalizing to the INPUT performed best, which yielded the highest percentage of exonic peaks and the GGAG motif compared to the other three methods (Figure 3). The protein level changes of Lin28b targets following Lin28b knockdown were also correlated with the relative enrichment of CLIP reads to INPUT (*R* = 0.79) [31], which further demonstrate the usefulness of normalizing CLIP reads to INPUT.

**Figure 4:**
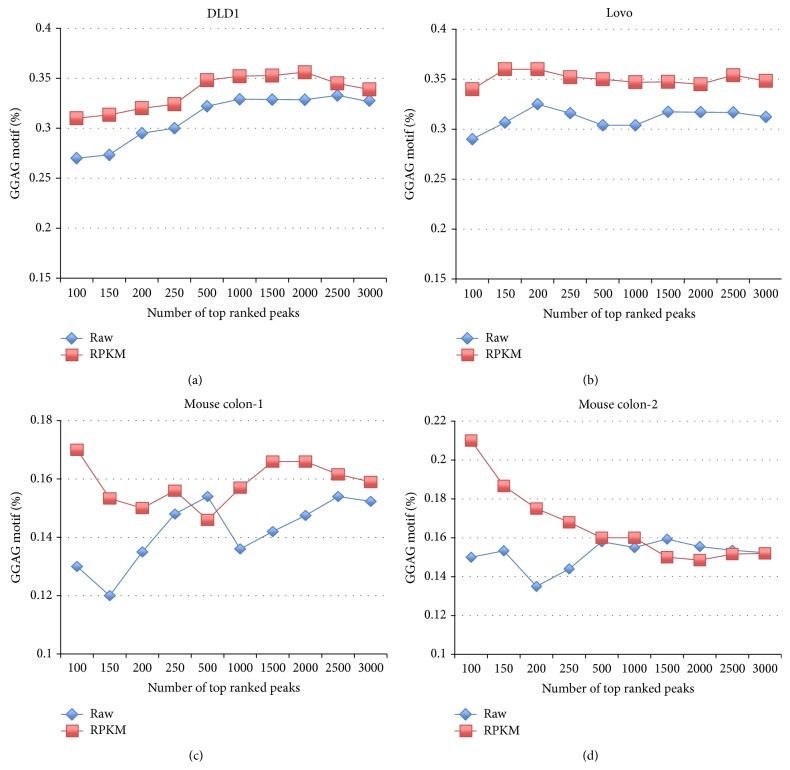
The percentage of peaks containing the GGAG motif for DLD1, Lovo, and mouse colon CLIP samples when peaks were ranked by reads coverage (Raw) or by relative enrichment to RPKM.

**Figure 5:**
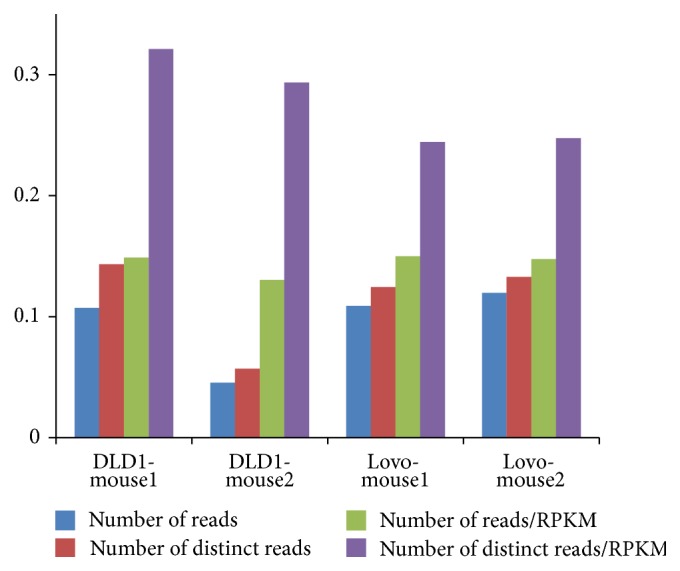
The correlation coefficient of peaks detected both in DLD1/Lovo and in mouse using different strategies.

For DLD1, Lovo, and mouse colon CLIP samples with the corresponding RNAseq as control, we compared ranking by the relative enrichment of CLIP reads to RPKM with the simple ranking by the reads coverage (Raw). Consistent with previous studies, ranking peaks by the relative enrichment of CLIP reads to RPKM performed better than the simple ranking method, which yielded higher percentage of the GGAG motif occurrence in DLD1, Lovo, and mouse colon samples (Figure 4). In addition to the motif occurrence, we also identified peaks common in both DLD1/Lovo and mouse samples, which are evolutionary conserved and can be considered as reliable Lin28b binding targets. The rank of binding targets in human and mouse would be correlated if the ranking could represent binding affinity. That is, the higher the correlation, the better the representation of binding affinity of the ranking strategy. We compared four different strategies, which included ranking by the coverage of all reads, distinct reads, the relative enrichment of all reads to RPKM, and the relative enrichment of distinct reads to RPKM (Figure 5). Consistent with our previous results, using distinct reads obtained higher correlation than using all reads. Furthermore, ranking peaks by the relative enrichment of distinct reads to RPKM obtained the highest correlation, ranging from 0.25 to 0.32. These results suggest that normalizing CLIP data to mRNAseq can improve the specificity when RBP targeting messenger RNA.

### 3.3. Motif Discovery

Motif discovery within CLIP peaks or surrounding regions reveals the unanticipated sequence signals associated with the RBP of interest. In a typical application of* de novo* motif analysis, motifs are generally discovered by differential enrichment analysis between sequences of peak regions and background sequences. Therefore, selection of an appropriate set of background sequences is very important. We enumerated all tetramers, ranked them by occurrence frequency, and filtered out insignificant ones. We used two different sets of background sequences to calculate the significance of enrichment. One was automatically generated by Homer where sequences were randomly selected from the genome with matched GC content [43], while the other was extracted from peaks identified from the INPUT samples. CTGG, GCTG, CCTG, TGGA, and TCCT were found to be the most frequently occurring tetramers in all Caco-2 CLIP samples; however, these motifs did not show any significant enrichment when compared to the background INPUT sequences (*P* value = 1) (Additional File 4). Previous studies have reported the excess of CTG and CT/TG on coding sequence structures [50], suggesting that the excessive recurrences of these motifs are mostly due to the coding features but not associated with Lin28b. Those false positives were successfully removed by differential motif analysis between target sequences and the background INPUT sequences. After filtering (FDR < 0.001), the motif GGAG ranked the first in all three Caco-2 replicate samples (Table 2 and Additional File 4), which provided validation of the success of using INPUT sequences as background. The method using randomly generated sequences from the reference genome as background reduced some degree of bias introduced by coding features, for example, filtering out the motif CCTG; however, it also removed the true motif GGAG. Since CLIP target regions are RNA, which generally has different sequence features from genomic regions, generating random sequences from the genome cannot define the background model correctly.

## 4. Discussion

Using Lin28b CLIP-Seq studies with input RNA or mRNAseq as control samples, we presented a systematic evaluation of different strategies to implement data preprocessing, peak normalization and ranking, and motif discovery for the analysis of CLIP-Seq data. We found that counting only distinct reads, normalizing to input RNA or mRNAseq, and defining the background model from control samples can improve the quality of binding sites. These findings will enable the design of refined experimental and computational protocols of CLIP-Seq studies.

To date, generating a high resolution and precise map of protein-RNA interactions still remains a big challenge, which requires novel experimental, computational, and statistical solutions. The crosslinking efficiency varies between proteins and the optimal protocol should be determined experimentally for individual proteins. Improving antibody specificity and optimizing the conditions of partial digestion with a relatively unspecific nuclease will help increase the library complexity and reduce the number of false positives. Recently, analysis of cross-linked induced mutation sites (CIMS) provides a nucleotide-resolution map of protein-RNA interactions [31, 51, 52]. Combining CLIP peaks with CIMS will improve the quantification of RBP binding. However, it is still far from fully understanding fundamental properties of cross-linking and its local sequence preference. Unlike Nova-CLIP and Ago-CLIP [51], which revealed deletions in ~8%–20% of CLIP reads, Lin28b-CLIP had very low percentage of reads containing deletions [31]. The percentage of insertion and substitution reads were similar between CLIP and input samples and cannot be used as CIMS signatures [31]. Therefore, the percentage of CIMS sites and the usefulness of each kind of CIMS signatures (insertion, deletion, and substitution) should be evaluated before CIMS sites are used. Novel bioinformatics methods to account for the local sequence features will help identify binding sites and reveal the binding motif accurately. Additionally, computational methods are needed to summarize scores of peaks in the given RNA and measure the effect of protein-RNA interactions. Finally, binding does not always suggest function. With more binding preferences of RBPs studied [53] and various kinds of omics data available [54], integrating CLIP-Seq with multiple omics data is necessary to reliably infer the functional effect of RBP binding events and to obtain a comprehensive view of posttranscriptional regulatory networks.

## 5. Conclusions

In this study we presented the first systematic comparison of different strategies to implement major CLIP-Seq data analysis steps. Our findings can serve as the practical guideline for CLIP experiments design and the comprehensive analysis of CLIP-Seq data.

## Supplementary Material

Additional File 1: Reads distribution of CLIP, input and RNA-seq samples.
Additional File 2: CLIP sequence alignment nearby “GGAG” motif within let-7d.
Additional File 3: Summary of peaks identified using distinct reads or all reads.
Additional File 4: The occurrence number of tetramers and the enrichment significance between CLIP and background sequences.

## Figures and Tables

**Table 1:** Mapping summary of CLIP, INPUT, and RNAseq reads.

Sample		Total reads	Aligned reads (%)	Unique aligned reads (%)
Human	Caco2_CLIP_1	34,498,894	12,095,664 (35.1%)	10,977,657 (31.8%)
	Caco2_CLIP_2	33,617,355	12,650,034 (37.6%)	11,514,201 (34.3%)
	Caco2_CLIP_3	48,866,964	17,965,486 (36.8%)	15,502,638 (31.7%)
	Caco2_INPUT_1	26,095,707	4,634,066 (17.8%)	3,257,784 (12.5%)
	Caco2_INPUT_2	40,683,388	7,954,926 (19.6%)	5,717,731 (14.1%)
	Caco2_INPUT_3	33,214,425	6,393619 (19.2%)	3,729901 (11.2%)
	DLD1_CLIP	36,860,853	18,303,689 (49.7%)	10,826,660 (29.4%)
	DLD1_RNAseq	196,664,529	120,668,419 (61.4%)	111,056,779 (56.5%)
	Lovo_CLIP	35,860,144	16,426,136 (45.8%)	8,435,054 (23.5%)
	Lovo_RNAseq	193,142,724	130,200,660 (67.4%)	120,721,857 (62.5%)

Mouse	Colon_CLIP_1	62,821,728	18,841,261 (30.0%)	13,884,667 (22.1%)
	Colon_CLIP_2	63,087,357	22,749,788 (36.1%)	15,226,733 (24.1%)
	Colon_RNAseq_1	56,706,471	44,663,179 (78.8%)	34,097,906 (60.1%)
	Colon_RNAseq_2	62,437,020	51,525,926 (82.5%)	40,559,464 (65.0%)

**(a):** 

Caco2-1				
Background from INPUT			Raw	Background from genome
4mers	Percent (%)	*P* value	Rank	*P* value
GGAG^a^	19.6^a^	1.36*e* − 07^a^	10	1.00
AGGA^b^	18.4^b^	8.61*e* − 13^b^	17	1.00
CAGG^c^	18.3^c^	4.52*e* − 20^c^	19	1.00
TGGC^d^	18.3^d^	4.54*e* − 04^d^	20	0.18
GTGG^e^	18.3^e^	4.96*e* − 12^e^	21	0.65
TGGG^f^	17.8^f^	3.65*e* − 12^f^	23	1.00
AAGA	17.0	4.72*e* − 09	28	0.05
GCAG^g^	17.0^g^	1.20*e* − 05^g^	29	1.00
GGTG	16.8	2.56*e* − 12	30	1.00
GGCT	16.8	2.11*e* − 07	32	1.00

**(b):** 

Caco2-2				
Background from INPUT			Raw	Background from genome
4mers	Percent (%)	*P* value	Rank	*P* value
GGAG^a^	20.1^a^	9.07*e* − 10^a^	9	1.00
TGGC^d^	19.1^d^	2.97*e* − 06^d^	13	0.05
GTGG^e^	18.8^e^	1.07*e* − 13^e^	16	0.41
TGGG^f^	18.4^f^	6.45*e* − 11^f^	19	1.00
GGTG	17.9	9.79*e* − 15	23	1.00
AGGA^b^	17.7^b^	8.42*e* − 09^b^	24	1.00
GCAG^g^	17.6^g^	3.70*e* − 11^g^	26	1.00
CAGG^c^	17.3^c^	1.00*e* − 14^c^	27	1.00
CAGA	17.0	1.27*e* − 07	30	1.00
AAGA	16.1	1.78*e* − 05	39	1.00

**(c):** 

Caco2-3				
Background from INPUT			Raw	Background from genome
4mers	Percent (%)	*P* value	Rank	*P* value
GGAG^a^	21.2^a^	1.05*e* − 32^a^	7	1.00
GAAG	19.5	1.98*e* − 11	12	7.21*e* − 11
TGGC^d^	19.4^d^	3.19*e* − 08^d^	13	3.22*e* − 04
CAGG^c^	18.3^c^	9.98*e* − 28^c^	18	1.00
AGGA^b^	17.9^b^	1.26*e* − 23^b^	21	1.00
TGGG^f^	17.9^f^	1.39*e* − 19^f^	22	1.00
GTGG^e^	17.8^e^	2.68*e* − 21^e^	24	1.00
GCAG^g^	17.7^g^	6.28*e* − 13^g^	26	1.00
AGAA	17.4	4.83*e* − 07	27	1.00
GGAA	17.1	9.00*e* − 05	28	1.00

^*^Tetramers common in all three samples have the same letters.
