# Supplementary material for: Assessing Computational Steps for CLIP-Seq Data Analysis
Source: Biomed Res Int. 2015 Oct 11;2015:196082. doi: 10.1155/2015/196082 (PMC4619761; doi:10.1155/2015/196082)
Supplement: Supplementary file 1 — Additional File 1: Reads distribution of CLIP, input and RNA-seq samples. Additional File 2: CLIP sequence alignment nearby “GGAG” motif within let-7d. Additional File 3: Summary of peaks identified using distinct reads or all reads. Additional File 4: The occurrence number of tetramers and the enrichment significance between CLIP and background sequences. [file 196082.f1.zip › additional file 1-3.pdf]

Additional File 1

Caco2_INPUT_1

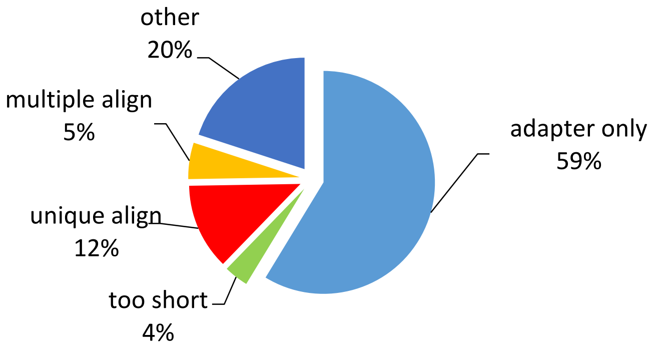

Caco2_CLIP_1

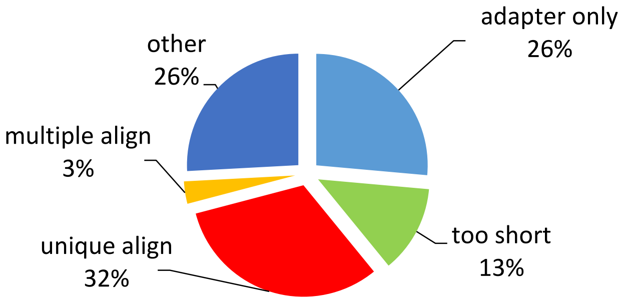

Caco2_INPUT_2

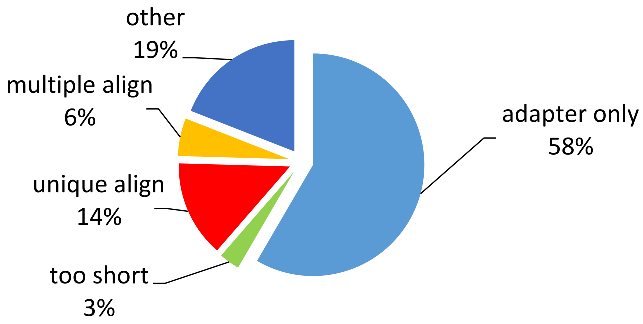

Caco2_CLIP_2

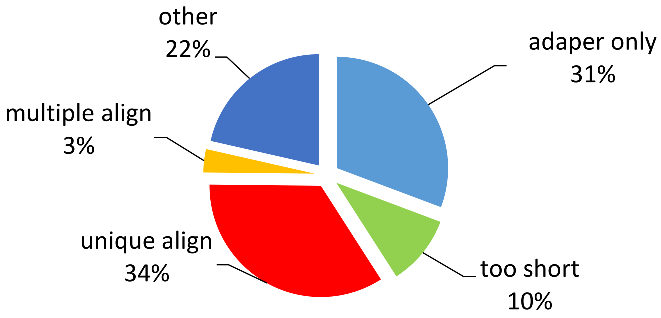

Caco2_INPUT_3

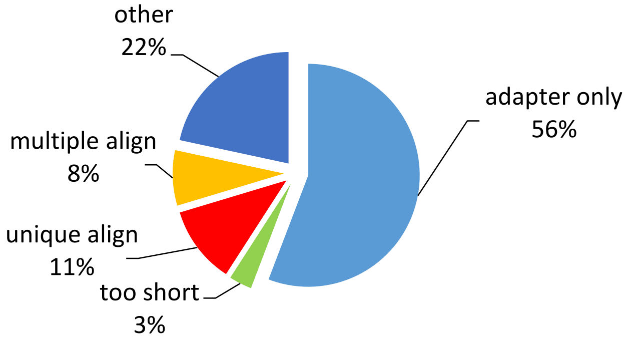

Caco2_CLIP_3

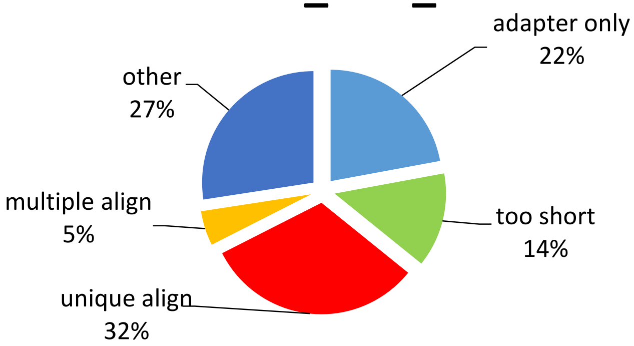

Additional File 2

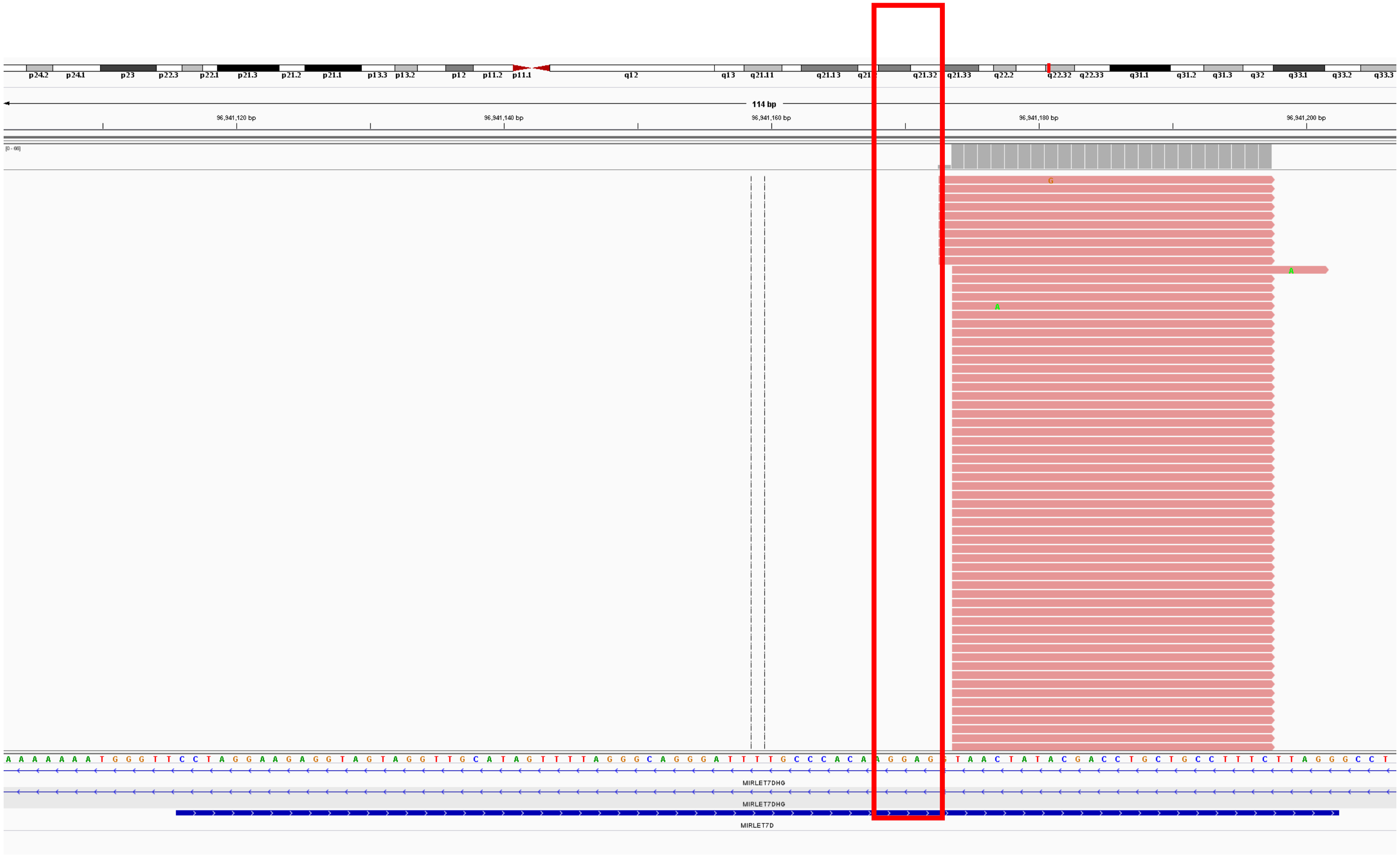

Additional File 3

		Caco2-1	Caco2-2	Caco2-3	DLD1	Lovo	Colon-1	Colon-2
Distinct reads	No. peaks	3875	4138	6832	17225	16780	8510	6781
	No. peaks in exon	3405	3265	4984	14326	15437	6658	4942
	Percent (%)	87.9	78.9	73.0	83.2	92.0	78.2	72.9
	No. peaks with GGAG motif	1137	1250	1984	4720	4266	1923	1464
	Percent (%)	29.3	30.2	29.0	27.4	25.4	22.6	21.6
All reads	No. peaks	4732	4376	7223	65548	80424	17580	13302
	No. exonic peaks	4078	3383	5159	43028	65571	11604	6630
	Percent (%)	86.2	77.3	71.4	65.6	81.5	66.0	49.8
	No. peaks with GGAG motif	1371	1300	2089	15720	18740	3839	2611
	Percent (%)	29.0	29.7	28.9	23.4	23.3	21.8	19.6
